# The Association between Vitamin D Hypovitaminosis and Cardiovascular Disease Risk in Saudi Diabetic Patients Type II

**DOI:** 10.1155/2022/6097864

**Published:** 2022-09-23

**Authors:** Abeer Ahmed Alrefai, Elsayed Elsalamony, Sameer H. Fatani, Zeinab A. Kasemy, Abdulaziz Fatani, Hala Fawzy Mohamed Kamel

**Affiliations:** ^1^Department of Medical Biochemistry and Molecular Biology, Faculty of Medicine, Menoufia University, Shebin El-kom 32511, Egypt; ^2^Department of Biochemistry, Faculty of Medicine, Umm Al-Qura University, Makkah 21955, Saudi Arabia; ^3^Internal Medicine Department, Endocrinology Unit, Mansoura University, Mansoura, Egypt; ^4^Department of Public Health and Community Medicine, Faculty of Medicine, Menoufia University, Shebine Elkoum 32511, Egypt; ^5^Faculty of Medicine, King Abdulaziz University, Geda, Saudi Arabia; ^6^Department of Medical Biochemistry and Molecular Biology, Faculty of Medicine, Ain Shams University, Cairo 11566, Egypt

## Abstract

We evaluated the prevalence and association of Vitamin D deficiency with glycemic control and CVD risk in T2DM patients. Serum 25 (OH)D_3_, lipid profile, glucose panel, HbA1c, serum insulin, and HOMA-IR were assessed in 93 T2DM patients and 69 controls. 10 years and lifetime ASCVD risk scores were calculated. The levels of 25(OH)D_3_ were significantly low in T2DM patients compared to the control. T2DM patients with hypovitaminosis D displayed significantly increased FBG, insulin, and HOMA-IR compared to normovitaminosis. Their lifetime and 10-year ASCVD risk scores were significantly higher regardless of vitamin D deficiency levels (*P*=0.006; *P*=0.023) in comparison to patients with sufficient levels of vitamin D. Among patients, the lifetime and 10 years of ASCVD risk showed a significant negative correlation with serum 25(OH)D_3_ and HDLc (*P*=0.037; 0.018) (*P*=0.0001), respectively, and significant positive correlation with T2DM duration, serum insulin, and HOMA-IR (*P*=0.018; 0.0001) (*P*=0.002; 0.001) (*P*=0.005; 0.001), respectively. The 10-year ASCVD risk exhibited a significant positive correlation with FBG (*P*=0.003) and HbA1c (*P*=0.009). T2DM duration was a predictor of vitamin D deficiency among T2DM patients (*β* = 0.22; CI = 0.002–0.04). There is a considerable association between lifetime and 10 years of ASCVD risk with hypovitaminosis D in T2DM, regardless of the deficiency levels which could be predicted by the diabetes duration.

## 1. Introduction

The deficiency of vitamin D has been identified lately as a major health concern [1]. As a multifunctional fat-soluble vitamin, consequences of deficiency of vitamin D might not only affect musculoskeletal metabolism and functions, but it will modulate many other cellular events, such as immunological response, cell proliferation and differentiation, insulin homeostasis, and metabolic functions as well [2]. Thus, it might play a vital role in the pathogenesis of insulin resistance, type 2 diabetes mellitus (T2DM), and cardiometabolic complications of T2DM patients [3]. By the effect of UV light, 7-dehydrocholesterol (provitamin D_3_) is converted into (cholecalciferol) vitamin D_3_ that is transported to the liver with dietary vitamin D_2_ to be hydroxylated by the enzyme P 450 vitamin D-25-hydroxylase into 25-hydroxyvitamin D. Furthermore, hydroxylation occurs in the kidney and to less extent in monocytes, placenta, and macrophages, by the enzyme (CYP27B1)25(OH)D31*α*-hydroxylase yielding 1,25-hydroxyvitamin D [1,25(OH)2D [25(OH)D_3_] [4].

There is growing evidence linking pathognomonic and molecular mechanisms of deficient vitamin D status with the development of T2DM, and it has been postulated that 1,25 dihydroxycholecalciferol or 1,25(OH)_2_D_3_ may increase insulin sensitivity, thus resulting in the cellular responsiveness for transportation of glucose [5]. Maestro and his colleagues identified vitamin D response elements (VDRE) in the promotor area of the insulin gene, which enhance insulin expression and consequently insulin sensitivity; therefore, binding of vitamin D to VDRE activates the transcription of the insulin receptor gene [6]. Alpha, 1, hydroxylase enzyme, that was thought to be expressed only by kidney cells, has been shown to be expressed too by islets of Langerhans containing beta cells of the pancreas that primarily regulate insulin secretion [7]. In addition, vitamin D reacts with its response elements at the promotor area of cytokine genes causing repression of the transcriptional factors involved in cytokine production and modulating their effects which could be linked to insulin resistance [8].

Vitamin D deficiency has been linked to several pathological events besides T2DM, such as obesity, dyslipidemia, endothelium dysfunctions, and hypertension [9]. Therefore, the role of vitamin D deficiency as a risk factor for cardiovascular diseases has attained the extensive interest of researchers; however, the pathological mechanisms for such interplay have not been fully understood [10].

Saudi Arabia has been ranked as the seventh worldwide and as the second middle east country for the incidence of T2DM [11]. Since recent reports indicated that vitamin D deficiency was abruptly increasing in the Saudi population to reach around 60% [12], hypovitaminosis D might potentiate the expected risk for cardiovascular diseases in T2DM patients that necessitate early identification and intervention of this problem [13]. Therefore, our aim in this study was to assess the prevalence and impact of vitamin D deficiency and insufficiency in T2DM patients and the possible implication of vitamin D levels on glycemic control and cardiovascular risk factors.

## 2. Subject and Methods

### 2.1. Study Design and Population

This study enrolled 93 T2DM patients, who were recruited from the Diabetes and Endocrinology clinic, at Al Noor hospital in Makkah according to the following criteria aged > 30 years, not pregnant or lactating, under dialysis or affected by other types of diabetes, chronic disease, or cancer. The control group comprised 69 healthy individuals who were matched for age and gender to the T2DM group. All subjects were exposed to detailed history-taking, especially focused on the duration of diabetes, smoking, medical history, and full clinical examination. The diagnosis of hypertension in the studied group was confirmed if the measurement of at least the systolic blood pressure (SBP) was 140 mmHg or more and/or the diastolic blood pressure (DBP) was 90 mmHg or more on two successive and separate occasions or if the patient was on antihypertensive treatment [14]. Anthropometric measurement was taken as weight in (Kg), height in (m), and body mass index (BMI) which was calculated as (Kg/m^2^). The BMI categories for normal, overweight, and obesity were 18–24.9, 25–29.9, and ≥30, respectively [15].

### 2.2. Blood Sampling

From each subject, two blood samples were collected, a morning fasting sample and another 2-h postprandial sample. A 7 ml of venous blood was withdrawn under complete aseptic condition. Then, the whole blood was placed into two sterile vacutainer tubes, one plain tube, and one EDTA tube. The EDTA tube was used for the assessment of whole blood HbA1c. The plain tube was centrifuged at 1500 rpm for 15 min; the resulting serum was stored at −20°C till further assessment of the studied parameters.

### 2.3. Laboratory Procedure

- The stored sample was utilized for assessing the following parameters using the standardized enzymatic colorimetric kits for fasting blood glucose (FBG) and 2-h postprandial glucose (2H-PPG) by the glucose oxidase method (spin react diagnostic kit, Girona, Spain), triglycerides (TG), total cholesterol (TC), low-density lipoprotein cholesterol (LDLc) and high-density lipoprotein cholesterol (HDLc) (Bio Merieux kit, France), urea, and creatinine (Diamond diagnostic kits, Germany), and C-reactive protein was assessed using standardized and automated assays.
- The glycemic control for each subject was evaluated by assessment of HbA1c in the whole blood sample via full automated high-performance liquid chromatography (HPLC) supplied by TecoDiagnostics, Lakeview Ave, Anaheim, CA, USA [16].
- Serum insulin levels were determined using the enzyme-linked immunosorbent assay (ELISA) (DRG International, USA), insulin resistance was assessed by homeostasis model assessment of insulin resistance (HOMA-IR) = fasting serum glucose (mg/dl) × serum insulin (uIU/mL)/405 [17].
- The levels of 25(OH)D3 were measured by the electrochemiluminescence assay on a Cobas immunoassay analyzer (Creative Biolab, Ramsey Road, Shirley, NY 11967, USA). Levels of 25-hydroxyvitamin D3 were stratified according to the classification of the Endocrine Society Clinical Practice Guidelines in 2011, the deficiency cut-off level was below 20 ng/mL, the insufficiency range was 20–29 ng/mL, and the adequate cut-off level was 30 ng/mL [1].
- For assessment of the cardiovascular disease risk in the studied groups, we used both the lifetime atherosclerotic cardiovascular disease risk (lifetime ASCVD) and the 10-year ASCVD risk assessment tools. For the calculation of the ASCVD score, the following data were included; demographic variables were included such as age and gender, besides the clinical and biochemical laboratory variants such as SBP, TC, HDLc, and history of diabetes, hypertension, or smoking. The calculated values for both risk scores were expressed as percentages. The optimal risk for lifetime ASCVD was estimated at cut off 5% for men and 8% for women, while the optimal risk for 10-year ASCVD was 1.2%, and patients with higher scores than 1.2% were at high risk for CVD development, whereas those with scores less than 1.2% were at low risk [18].

### 2.4. Sample Size

A statistical power analysis was performed after sample size estimation, based on data from the current study (*N* = 162), comparing the diabetic group to controls. The effect size (ES) for 25(OH)D_3_ in this study was 0.59, considered to be moderate using Cohen's (1988) criteria, with an alpha = 0.05 and sample size = 93 in the T2DM group and 69 in controls, a posthoc power analysis was conducted with these effect sizes (G Power 3.1), and it was approximately (1−_*β*_) = 0.962 [19]. Thus, the power analysis for a sample size of 162 was adequate for the main objective of this study.

### 2.5. Statistical Analysis

IBM SPSS software package version 16.0 was used to analyze the data. The normality of the distribution of variables was verified by the Kolmogorov–Smirnov test, and categorical variables were assessed using the chi-square test. For normally distributed quantitative variables, Student's *t*-test and ANOVA test with posthoc test were used, while the Mann–Whitney test and K test were used for not normally distributed quantitative variables. The linear regression analysis test was used to evaluate predictors of hypovitaminosis. The significance of the obtained results was estimated at the 5% level.

## 3. Results

### 3.1. Demographic, Clinical, and Biochemical Data among Participants

Table 1 illustrated demographic, clinical, and biochemical data among participants, who were matched for age (*P*=0.15) and sex (*P*=0.42). Concerning anthropometric measures of all participants, the BMI was significantly higher in T2DM patients (32.57 ± 5.16 kg/m^2^) as compared to controls (29.28 ± 2.09 kg/m^2^) (*P*=0.001); 74.2% of T2DM patients were obese, 24.7% were overweight, and 1% was normal weight vs. 47.8%, 43.5%, and 8.7% in the control group (*P*=0.001), respectively. The median of the duration of T2DM diagnosis was 4 years. As expected, the FBG, 2H-PPG, HbA1C, insulin, HOMA-IR, ESR, CRP levels, and blood pressure were significantly higher among patients with T2DM in comparison to the control subjects (*P*=0.0001). 72% and 42% of the T2DM patients showed HbA1c > 7.5% (poor glycemic control) and were hypertensive (not shown). A significant increase in TC, LDLc, TG, and decreased HDLc levels were noted in T2DM patients compared to the control group (*P*=0.0001), (*P*=0.01), (*P*=0.008), and (*P*=0.0001), respectively. Consequently, LDLc/HDLc was significantly higher in T2DM patients compared to the control group (*P*=0.0001).

### 3.2. Vitamin D Status among Participants

The median of 25(OH)D_3_ level was significantly low in T2DM patients as compared to the control group (18 Vs. 21) (*P*=0.0001). The overall prevalence of 25(OH)D_3_ status among patients was 51.6%, 26,9%, and 21.5 for deficiency, insufficiency, and sufficiency versus 40.6%, 29%, and 30.4% for control subjects.

### 3.3. Normovitamin D T2DM Vs. Those with Hypovitaminosis

The demographic, clinical, and biochemical data among T2DM patients with vitamin D hypovitaminosis versus those with sufficient levels were shown in Table 2. 78.5% of the T2DM patients were hypovitaminosis, and 21.5% of those patients were normovitaminosis. The overall prevalence of hypovitaminosis D was more common among males (60.3%) (*P*=0.02) and obese diabetics (79.5%) (*P*=0.035). The management plan revealed a nonsignificant difference (*P*=0.4) between T2DM regarding 25(OH)D_3_ status Table 2. T2DM patients with hypovitaminosis displayed significant differences regarding FBG (*P*=0.0001), serum insulin (*P*=0.01), and HOMA-IR (*P*=0.001) compared to those with sufficient D levels but not for the HbA1c level (0.3). Furthermore, TG and LDLc levels were significantly higher in T2DM patients with hypovitaminosis 25(OH)D_3_ as compared to those with sufficient 25(OH)D_3_ levels (*P*=0.045) and (*P*=0.04), respectively.

### 3.4. ASCVD Risk in T2DM Related to Vitamin D Status

The frequency of 10 years of ASCVD risk among T2DM patients with hypovitaminosis was 54.8% vs. 5% in those with normal 25(OH)D_3_ levels. For the CVD risk score, T2DM patients with hypovitaminosis revealed significantly increased scores of lifetime and 10-year ASCVD risk when compared to those with sufficient 25(OH)D_3_ level (*P*=0.006) and (*P*=0.023), respectively. Furthermore, T2DM patients with 25(OH)D_3_ insufficiency and deficiency revealed significantly increased scores for lifetime ASCVD risk when compared to those with 25(OH)D_3_ sufficiency (*P*=0.04; 0.01), respectively. Moreover, T2DM patients with 25(OH)D_3_ deficiency exhibited a significant increase of 10 years of ASCVD risk as compared to those with a sufficient level (*P*=0.012) Table 3.

### 3.5. Correlation of ASCVD Risk with Variables in T2DM

Among T2DM patients, the lifetime and 10 years of ASCVD risk showed significant negative correlation with serum 25(OH) D_3_ (*r* = −0.22, *P*=0.037); (*r* = −0.25, *P*=0.018) and HDLc (*r* = −0.37, *P*=0.0001); (*r* = −0.42, *P*=0.0001) respectively and significant positive correlation with body weight (*r* = 0.33, *P*=0.019); (*r* = 0.26, *P*=0.0001), T2DM duration (*r* = 0.24, *P*=0.019); (*r* = 0.42, *P*=0.0001), serum insulin (*r* = 0.32, *P*=0.002), (*r* = 0.35, *P*=0.001), HOMA-IR (*r* = 0.29, *P*=0.005), (*r* = 0.34, *P*=0.001), TC (*r* = 0.26, *P*=0.012), (*r* = 0.4, *P*=0.0001), and LDLc (*r* = 0.26, *P*=0.01), (*r* = 0.25, *P*=0.016). In addition, 10-year ASCVD risk exhibited a significant positive correlation with FBG (*r* = 0.3, *P*=0.003), 2H-PPG (*r* = 0.22, *P*=0.03), and HbA1c (*r* = 0.27, *P*=0.009) Table 4.

### 3.6. Regression Analysis of Hypovitaminosis in T2DM

Linear regression analysis showed that T2DM duration was a predictor of 25(OH)D_3_ deficiency among patients (*P*=0.03) Table 5.

## 4. Discussion

In the current study, the median level of 25(OH)D_3_ was significantly low in T2DM patients in comparison to the control group. Several studies in various ethnic groups were in agreement with our findings [20, 21]. Pietschmann and his colleagues stated that 25(OH)D_3_ levels were significantly decreased in T2DM but not in T1DM patients when compared with healthy controls [22]. Vitamin D has been claimed of being a contributor to the pathogenesis of T2DM so that vitamin D deficient T2DM patients might be encountered the enhancing effect of microvascular complications and worst glycemic control; thus, CVD has been evidently associated with vitamin D hypovitaminosis in T2DM patients as well [23].

In the current study, 25(OH)D_3_ status was assessed via measurement of serum 25(OH)D_3_ levels for all studied groups as its clearance rate is slower than 1,25(OH)2D, so the former is a preferable biomarker for vitamin D status, while the latter is clinically useful for follow-up and monitoring of certain genetic or acquired conditions linked to calcium, phosphate, or vitamin D [24]. Our results indicated that the overall prevalence of 25(OH)D_3_ status among T2DM patients was 51.6%, 26,9%, and 21.5% for deficiency, insufficiency, and sufficiency versus 40.6%, 29%, and 30.4% for control subjects, 25(OH)D_3_ hypovitaminosis was higher among the T2DM group (78.5%) than control subjects (69.6%); however, it did not reach a significant level. Most T2DM patients with hypovitaminosis (86%) were among the age group of (45–55 years). In agreement with our findings, a previous study in North India reported a prevalence of 81% vitamin D deficiency among T2DM patients [25]. Similarly, in Saudi Arabia, a meta-analysis reported 63.5% as an overall prevalence among 20,787 T2DM patients [12]. In another study in Jazan city in the south of Saudi Arabia, the prevalence was reported to be 60.8%, which is also lower than our reported prevalence value; however, both studies reported a significantly higher prevalence of vitamin D deficiency among the same age group of the current study (54.2 ± 11.8 years) [26]. Despite the agreement of most of these studies on the high prevalence of hypovitaminosis D in Saudi Arabian T2DM patients, however, there was considerable variability in their values among various regions of the Kingdom. The inconsistency of these results may be caused by the difference in assessment methodology used at various laboratories such as the reported 23% falsely positive results of 25(OH)_2_D_3_ when the chemiluminescence immunoassay was used [27]. Evermore, the cutoff value for defining each phase of vitamin D deficiency status has been extensively debated, yet most studies and recommendations rely on defining vitamin D insufficiency at 20–30 ng/ml, deficiency at less than 20 ng/ml, and the sufficient level at above 30 ng/ml, [1] which are the same cut off levels as the values we use in our study.

In this study, T2DM patients with hypovitaminosis 25(OH)D_3_ displayed significantly higher levels of FBG, serum insulin, HOMA-IR, TG, and LDLc compared to those with sufficient 25(OH)D_3_ levels. HbA1c levels were higher as well; however, they did not reach a significant value. In agreement with these findings, several studies reported that the levels of 25(OH)D_3_ were inversely correlated with impairment of glucose homeostasis and insulin resistance [28], HOMA-IR, TC, and HbA1c [29], whereas 25(OH)D_3_ levels were found to be related to glycemic control in T2DM [30]. In a previous report, patients with poor glycemic control displayed lower levels of serum 25(OH)D_3_ levels as compared to those with good glycemic control [31]. An inverse association was also reported between vitamin D serum levels and FBG levels and insulin resistance [32]. These findings might point to the potential causal relationship of hypovitaminosis 25(OH)D_3_ with T2DM or suggest that deficiency of vitamin D might be the result of diabetes, not the cause. Thus, being a cause or consequence, hypovitaminosis vitamin D is strictly associated with poor glycemic control [33].

In fact, vitamin D affects pancreatic beta cell function and insulin secretion and resistance directly and indirectly via genomic and nongenomic cellular events. Directly, vitamin D induces the release of insulin from beta cells of the pancreas via binding to its specific vitamin D receptors (VDR) as its responsive elements have been identified at the promotor region of the insulin receptor gene. Indirectly, vitamin D contributes to insulin release from beta cells through regulation of calcium flux as well as extracellular calcium to ensure an adequate calcium pool [34], and such effect could be mediated by regulating the calcium-binding protein “calbindin” which controls the rate of release of insulin from pancreatic beta cells [35]. In a systematic review and meta-analysis, it has been speculated that a deficiency of vitamin D might affect the harmonizing mechanisms of extracellular and intracellular calcium and consequently hinder glucose-mediated insulin release from beta cells [36]. Thus, vitamin D might have a pivotal role in improving insulin resistance in T2DM patients and raising the sensitivity of peripheral insulin in patients with impaired glucose tolerance.

In the current study, T2DM patients with 25(OH)D_3_ deficiency levels (<20 ng/ml) revealed significantly increased both lifetime and 10-year ASCVD risk scores when compared to those with a sufficient 25(OH)D_3_ level (≥30 ng/ml); moreover, T2DM patients with 25(OH)D_3_ insufficiency (20–29 ng/ml) showed only a significantly increased lifetime ASCVD risk score in comparison to those with sufficient 25(OH)D_3_ levels. The studied cohort with hypovitaminosis 25(OH)D_3_ also displayed significantly increased serum insulin, LDLc, and HOMA-IR with poor glycemic control indices as FBG compared to T2DM patients with adequate 25(OH)D_3_ levels regardless of the management modalities whether oral therapy, insulin, or combined. Considering these findings altogether, they might point toward the possible atherogenic role of 25(OH)D_3_ hypovitaminosis in the development of CVD in poorly controlled T2DM patients. In line with our finding, Alaidarous and his colleagues found that hypovitaminosis of vitamin D is significantly associated with CVD risk in T2DM patients especially in uncontrolled cases regardless of the duration of the disease; however, they used different assessment units of vitamin D deficiency as a cut off (<50 nmol/l) which is equivalent to the cut off we used in our study (<20 ng/ml) [37].

Recently, the rise of serum levels of vitamin D is found to be associated with improvement of glucose tolerance and better control of diabetes; each rise of 1 ng/ml of vitamin D levels is associated with the decline of HbA1c percentage by 0.09%, signifying the key value of the rise of vitamin D in declining the risk of CVD complications in T2DM patients [3].

Among the T2DM patients, the lifetime and 10 years of ASCVD risk showed a significant inverse correlation with serum 25(OH)D_3_ and HDLc, while both risk scores exhibited a direct significant correlation with weight, duration of T2DM, fasting insulin level, HOMA-IR, LDLc, and TC. Linear regression analysis showed that T2DM duration was a significant predictor of vitamin D deficiency in T2DM patients. Along the same line as our findings, Alaidarous and his colleagues reported a converse correlation between vitamin D levels with lifetime ASCVD risk scores in uncontrolled patients with long duration of diabetes but a positive association with newly-diagnosed uncontrolled T2DM patients [37]. In controversy, a nonsignificant association has been reported between vitamin D levels and the duration of diabetes [38, 39].

Numerous studies and metanalysis have recently pointed to the complex connection of hypovitaminosis D with obesity, diabetes, hypertension, and endothelium dysfunction which explains the possible impact of low levels of vitamin D as a risk factor for CVD in T2DM patients [10, 40–43]; however, the underlying linking mechanisms have not been fully elicited. The proposed mechanisms include the VDR-mediated negative effect of vitamin D on the renin-angiotensin system, suppressing renin gene and consequently decreasing levels of renin [44]. Another mechanism, that might be involved, is the possible role of vitamin D in the downregulation of cytokines and anti-inflammatory mediators post-transcriptionally [45], thus potentiating immunological activation which is linked to atherosclerotic changes and the risk of CVD in hypovitaminosis D [13]. The above mechanisms might explain the superimposed role of low levels of vitamin D with the preexistence of hypertension in T2DM patients to increase the risk of the development of CVD [46]. It has been suggested by other interventional studies that vitamin D supplementation might enhance the secretion and sensitivity of insulin in patients with vitamin D deficiency [36, 47], thus causing significant beneficial effects on the CVD risk biomarkers [48]. Taking it altogether, our findings highlight the importance of early detection of vitamin D deficiency in T2DM patients especially those with poor glycemic control and a long duration of disease. In addition, our results emphasize the proposed pathognomonic mechanisms explaining the consequence of hypovitaminosis of vitamin D in increasing risk for CVD that might direct future studies to investigate the potential beneficial value of vitamin D supplementation as protective therapy against CVD morbidity and mortality [49].

The prevalence of 25(OH)D_3_ deficiency was high in Saudi patients with T2DM mainly among the age group of (45–55 y), predicted by the duration of T2DM, regardless of the treatment strategy. In addition, there was a direct association of lifetime and 10 years of ASCVD risk with 25(OH)D_3_ hypovitaminosis in T2DM. Moreover, there is an inverse relationship between FBG, HOMA-IR, and 25(OH)D3 status which account for the 10 years of ASCVD risk solely. Thus, maintaining adequate vitamin D status and avoiding deficiency may help to prevent CVD complications among patients with T2D. Furthermore, it is valuable to study if vitamin D should be given to only vitamin D deficient patients or all T2DM patients.

The present study had a few limitations. Firstly, a causal relationship cannot be established due to the cross-sectional study design. Further studies are required to evaluate the causative relationships between 25(OH)D_3_ and CVD, the effect of vitamin D supplementation on CVD, and T2DM patients. Secondly, this study is limited to Asian people, so our findings may not be applicable to other ethnic populations with different nutritional and behavioral aspects. Finally, the observational nature of the data and all these factors may affect the generalizability of this study.

## 5. Conclusion

25(OH)D_3_ level was significantly low in T2DM patients as compared to the control group, and there is a considerable association between lifetime and 10-year ASCVD risk with 25(OH)D_3_ hypovitaminosis in T2DM, regardless of the deficiency levels which could be predicted by the diabetes duration, revealing the importance of early detection of vitamin D deficiency and supplementation.

## Figures and Tables

**Table 1:** Demographic, clinical and biochemical data among studied groups.

Variables	Diabetic patients	Control group	
	(93)	(69)	
Age (years)	50.59 ± 6.03	49.39 ± 3.96	0.15
Gender			
Male	50 (53.8%)	42 (60.9%)	0.42
Female	43 (46.2%)	27 (39.1%)	
Smokers	10 (10.8%)	12 (17.4%)	0.16
Duration (years)	4 (1–24)	—	
Weight (Kg)	88.07 ± 17.55	81.17 ± 5.86	0.002^*∗*^
BMI (Kg/M^2^)	32.57 ± 5.16	29.28 ± 2.09	0.001^*∗*^
BMI categories			
18–25	1 (1%)	6 (8.7%)	0.001^*∗*^
25–29	23 (24.7%)	30 (43.5%)	
>30	69 (74.2%)	33 (47.8%)	
SBP (mmHg)	143.12 ± 24.73	117.61 ± 5.61	0.0001^*∗*^
DBP (mmHg)	89.03 ± 8.88	79.13 ± 4.8	0.0001^*∗*^
TC (mg/dl)	176.7 ± 34.81	143.8 ± 9.7	0.0001^*∗*^
TG (mg/dl)	142 (90–400)	128.3 (67–299)	0.008^*∗*^
LDLc (mg/dl)	105.77 ± 25.88	97.6 ± 14.3	0.01^*∗*^
HDLc (mg/dl)	40.7 ± 8.88	77.78 ± 13.1	0.0001^*∗*^
LDLc/HDLc	2.76 ± 1.06	1.29 ± 0.28	0.0001^*∗*^
FBG (mg/dl)	151.2 ± 39.65	87.47 ± 7.07	0.0001^*∗*^
2H-PPG (mg/dl)	205.4 ± 50.36	116.26 ± 19.4	0.0001^*∗*^
HbA1C (%)	8.01 ± 1.4	4.99 ± 0.39	0.0001^*∗*^
Insulin (uIU/ml)	16 (6–54)	11 (10–14)	0.0001^*∗*^
HOMA-IR	5.48 (2.1–20.77)	2.52 (1.98–3.18)	0.0001^*∗*^
CRP (mg/dl)	14.02 ± 4.67	3.04 ± 0.75	0.0001^*∗*^
ESR (mm/H)	19 (6–118)	11.9 (9.8–14.2)	0.0001^*∗*^
Vitamin D (ng/ml)	18 (6–34)	21 (12–34)	<0.001^*∗*^
Vitamin D status			
Sufficiency	21.5 (20)	30.4 (21)	0.3
In-sufficiency	26.9 (25)	29 (20)	
Deficiency	51.6 (48)	40.6 (28)	

^*∗*^statistically significant at *P* < 0.05.

**Table 2:** Variable of T2DM with hypovitaminosis D vs. nonhypovitaminosis.

Variables	Hypovitaminosis		*P*
	Yes *N* (%)	No *N* (%)	
	73 (78.5)	20 (21.5)	
Age (years)	50.7 ± 5.4	49.9 ± 8.1	0.6
Gender			
Male	44 (60.3)	6 (30)	0.016^*∗*^
Female	29 (39.7)	14 (70)	
Smokers			
	10 (13.7)	0 (0)	0.07
		
Duration (years)	4 (1–24)	5.5 (1–10)	0.28
Weight (Kg)	89.9 ± 18.1	81.4 ± 13.5	0.4
BMI (Kg/M^2^)	32.8 ± 5.1	31.4 ± 5.2	0.2
BMI category			
N (%)			0.035^*∗*^
18–25	2 (2.7)	0 (0)	
25–29.9	13 (17.8)	9 (45)	
>30	58 (79.5)	11 (55)	
SBP (mmHg)	143.7 ± 24.4	141 ± 26.4	0.6
DBP (mmHg)	89.3 ± 9.1	88 ± 7.8	0.5
FBG (mg/dl)	157.1 ± 41.3	129.7 ± 23.2	0.0001^*∗*^
2-HPPG (mg/dl)	206.9 ± 48.5	199.9 ± 57.6	0.6
HbA1c (%)	8.08 ± 1.4	7.7 ± 1.4	0.3
Insulin (uIU/ml)	17 (6–54)	12 (8–54)	0.01^*∗*^
HOMA-IR	5.6 (2.9–20.8)	3.8 (2.01–16.7)	0.001^*∗*^
Tc (mg/dl)	179.8 ± 34.3	165.5 ± 35.1	0.1
TG (mg/dl)	150 (90–400)	119.5 (92–334)	0.045^*∗*^
HDLc (mg/dl)	40.2 ± 8.6	42.8 ± 9.9	0.2
LDLc (mg/dl)	108.4 ± 26.6	96.3 ± 21.1	0.04^*∗*^
10-years ASCVD OR	54.8 (40)	5 (25)	0.016^*∗*^
Lifetime ASCVD score	54.7 ± 11.5	47.5 ± 9.1	0.006^*∗*^
10-years ASCVD score	9.6 (1–37.7)	4.2 (0.6–20.4)	0.023^*∗*^
Management			
OHA	20 (27.4)	5 (25)	
Oral combined	16 (21.9)	6 (30)	
OHA & insulin	23 (31.5)	3 (15)	0.4
Insulin	14(19.2)	6 (30)	

^*∗*^statistically significant at *P* < 0.05.

**Table 3:** Lifetime and 10-year ASCVD risk among type 2 diabetic patients in relation to serum vitamin D levels.

Vitamin D status	Lifetime ASCVD risk	*P*	10 years of ASCVD risk	*P*
T2DM (93)				
Sufficiency (20)	47.5 ± 9.1	0.028^*∗*^	4.05 (0.6–20.7)	0.027^*∗*^
Insufficiency (25)	54.3 ± 11	Post-hoc test	5.8 (2–29.3)	Mann-Whiteny test
Deficiency (48)	54.8 ± 11.8	*P * ^a^ = 0.04^*∗*^	9.6 (1–32.8)	*P* ^″^=0.012^*∗*^
		*P * ^b^ = 0.01^*∗*^		
		*P * ^c^ = 0.8		

Lifetime ASCVD risk by ANOVA, posthoc test: *P*^a^ : sufficiency vs. insufficiency, *P*^b^ : sufficiency vs. deficiency, *P*^c^ : insufficiency vs. deficiency. 10 years of ASCVD risk by Kruskall Whalis Mann–Whiteny test: *P*″: sufficiency vs. deficiency ^*∗*^ statistically significant at *P* < 0.05.

**Table 4:** Correlation of lifetime and 10-year ASCVD risk with variables among T2DM.

Variables	Lifetime ASCVD risk		10-year ASCVD risk	
	*r*	*P*	*r*	*P*
Weight	0.33	0.019^*∗*^	0.26	0.0001^*∗*^
T2DM duration	0.24	0.019^*∗*^	0.42	0.0001^*∗*^
HbA1c	0.12	0.24	0.27	0.009^*∗*^
FBG	0.06	0.5	0.3	0.003^*∗*^
2H-PPG	0.13	0.23	0.22	0.03^*∗*^
Insulin	0.32	0.002^*∗*^	0.35	0.001^*∗*^
HOMA-IR	0.29	0.005^*∗*^	0.34	0.001^*∗*^
TC	0.26	0.012^*∗*^	0.4	0.0001^*∗*^
LDLc	0.26	0.01^*∗*^	0.25	0.016^*∗*^
HDLc	−0.37	0.0001^*∗*^	−0.42	0.0001^*∗*^
Vitamin D	−0.22	0.037^*∗*^	−0.25	0.018^*∗*^

^*∗*^statistically significant at *P* < 0.05, *r*: Spearman's coefficient.

**Table 5:** Linear regression analyses to identify predictors of vitamin D status among T2DM.

Variables	Vitamin D deficiency		
	*β*	*P*	95% CI
T2DM duration	0.22	0.03^*∗*^	0.002–0.04

^*∗*^statistically significant at *P* < 0.05. CI : confidence interval.

## Data Availability

All the data are available if needed.
